# A Multiple Case Series Analysis of Six Variants of Attentional Bias Modification for Depression

**DOI:** 10.1155/2013/414170

**Published:** 2013-03-21

**Authors:** Anne-Wil Kruijt, Peter Putman, Willem Van der Does

**Affiliations:** ^1^Institute of Psychology, Leiden University, Wassenaarseweg 52, 2333 AK Leiden, The Netherlands; ^2^Department of Psychiatry, Leiden University Medical Center, 2333 ZA Leiden, The Netherlands; ^3^Leiden Institute of Brain and Cognition, 2333 ZA Leiden, The Netherlands

## Abstract

*Background*. Attention bias modification (ABM) is a new treatment for affective disorders. A meta-analysis of ABM for anxiety disorders showed that the effect size may be large but the number of studies is low. The working mechanism is still unclear, and little is known about the optimal treatment parameters. ABM for depression is much less studied. A few studies claimed positive effects but the sample sizes are low. Furthermore, the treatment parameters varied widely and differed from the anxiety literature. *Aim*. To select the most promising version of ABM for depression for further evaluation in clinical trials. *Methods*. Multiple case series design. We tested six versions of ABM that varied on stimulus duration and training direction. Thirty students with mild to moderate symptoms of depression underwent four sessions of ABM. Change of attentional bias was measured during each session. Generalization of treatment effects and the role of awareness of receiving training were also investigated. *Results*. None of the investigated versions of ABM had a consistent effect on attentional bias. Changes of attentional bias in individual participants the effects did not generalize to untrained stimuli. *Conclusion*. It is unlikely that any of these ABM versions will have a specific effect on symptoms in controlled studies.

## 1. Introduction

A growing body of literature reports that computerized attention training programs are successful in reducing symptoms of anxiety and depression. These attentional bias modification (ABM) procedures intend to reduce or even reverse patients' habitual tendency to direct their attention towards negative information. Most of these studies use variants of the dot probe task [1]. In ABM variants of this task, participants are implicitly taught to direct their attention away from negative information (a picture or word). This is accomplished by administering several hundreds of trials that involve brief simultaneous presentations of negative and neutral information, followed by a target. This target is systematically presented at the location of the neutral information [2]. In a recent meta-analysis, an effect size of .61 was reported for ABM for anxiety over a placebo training [3]. The authors concluded that these training programs show promise as a new treatment but also qualify the research on ABM as immature. Nine of the twelve studies were conducted in nonclinical samples (anxious students or adults), while only three studies concerned patients with an anxiety disorder. The effect sizes across trials also varied substantially, and an asymmetrical funnel plot was found, indicating a possible publication bias. Furthermore, it remains unclear whether the treatment actually exerts an effect on attentional bias to threat, the presumed mechanism of action. Actual bias chances are not always reported. Mediation analyses, if performed, give equivocal results, and in one study participants who were trained to direct attention towards and away from threat showed comparable reduction of emotional reactivity [4]. Several other unresolved questions remain regarding the optimal ABM design. Studies are needed that systematically vary variables such as stimulus durations, stimulus content, and sessions durations to establish the optimal training parameters [5].

 Currently and remitted depressed patients show attentional bias for sad words and faces (reviews: [6, 7]) and, compared to healthy controls, lack a bias towards positive information [8, 9]. Such biased information processing is theorized to play a role in the aetiology and maintenance of major depressive disorder [10]. Two studies to date, therefore, have tested ABM procedures in depression. Four sessions of ABM over the course of two weeks had no immediate effect on depression scores of 34 dysphoric students; however, a significant difference was observed between the ABM-treated and control groups at two weeks of followup [11]. A mediation analysis that supported the proposed working mechanism, the reduction of attentional bias for negative information over the course of treatment, was associated with eventual reduction in symptomatology. Attentional bias at followup, however, was not reported. Moreover, attrition was very high at 47%, rendering the conclusion that the treatment had the intended effect premature. In another paper [12] two studies were presented that tested the effects of an ABM procedure based on the emotional spatial cueing task, which is closely related to the dot probe task. The treatment did not affect attentional bias in depressed patients nor did it affect their symptom severity. In a dysphoric student sample, treatment also had no effect. In a post hoc analysis, however, symptom severity was decreased in a mildly dysphoric subgroup, whereas increased symptoms were observed in those with moderate to severe symptoms at baseline.

 In summary; ABM treatment for anxiety disorders has been called promising, but a close look at the individual clinical trials reveals many open questions, while ABM for depression is even less studied. Two studies have claimed small effects in nonclinical samples including an adverse effect in some participants. The ABM parameters used in both studies differ substantially from each other and from those typically used in anxiety disorder studies. Instead of carrying out another randomized controlled trial with one of these treatments, we propose that intensive case studies are needed to select the most promising approaches for evaluation in clinical trials. The current study is such a case study in which the effect of two parameters of the dot probe ABM paradigm, stimulus duration and aiming at inducing towards positive information versus reducing bias towards negative information, will be evaluated in a dysphoric student sample.

The depression ABM procedure designed by Wells and Beevers [11] differs from ABM procedures for anxiety disorders. Sad (depression relevant) pictures were used rather than threatening stimuli, as well as exceptionally long stimulus durations (3000 ms for faces and 4500 ms for scenic pictures). The spatial cueing ABM procedure introduced by Baert and colleagues also used a relatively long cue duration of 1500 ms [12]. The rationale is that, compared to anxiety, attentional biases in depression may be more pronounced at longer stimulus durations, after more elaborated processing and greater activation of relevant schemata [13]. A recent meta-analysis, however, found similar effect sizes for studies assessing attentional bias in depression, dysphoria, or induced dysphoria at 500 ms stimulus duration (*d* = 0.54, 7 studies) and at 1000 ms or higher (*d* = 0.59, 9 studies) [6]. 

 In addition to bias towards sad information, remitted and currently depressed patients also show a reduction in bias towards happy faces compared to healthy controls [8, 9]. This combination of biases was also found in dysphoric students [14] and girls at risk for depression [15]. The spatial cueing ABM procedure tested by Baert and colleagues was designed to induce bias towards positive information as well as reduce bias towards negative information [12]. One other study, that did not explicitly target nor measure depression symptomatology, successfully modified attentional bias using a dot probe ABM procedure to induce bias towards positive word stimuli in healthy students at a stimulus duration of 500 ms [16]. Eye-tracking revealed that ABM reduced the time participants spent gazing at negative portions of images.

 In the present study, 30 students with mild to moderate symptoms of depression engaged in one of six variants of the ABM paradigm for four sessions within a week. Each condition was defined by a combination of one of two training directions, away from neutral towards happy information or away from sad towards neutral information, and either a long, a short, or a variable stimulus duration. To our knowledge such a variable duration has not been used before. The rationale is that, instead of training participants to direct their attention at a specific point in time, a variable stimulus duration will train participants at several stages of attention orienting simultaneously. We used an 85% congruency contingency, that is, 85% of the trials were congruent or incongruent trials, dependent on the training direction. This allowed monitoring attentional bias per session and also obscured the rationale of the study from the participants [11]. Attentional bias was assessed throughout the four training sessions and at pre- and posttraining using a separate stimulus set. Upon completion of the training procedure, participants were interviewed regarding their awareness of the task mechanics, as unpublished data suggest that training may only have an effect on symptoms if participants remain unaware of the congruency contingency [17].

 In summary, we tested the effects of six variants of an ABM procedure in dysphoric students. Four treatment sessions were conducted over the course of one week. The main outcome measure was the change of attentional bias, assessed during the training as well as posttraining on a separate set of stimuli. Symptom changes were not expected within this time frame. We hypothesized that attentional bias would change in the intended direction during the course of treatment and that this change would be generalized to untrained stimuli. The effect of participants' awareness of receiving training was also explored.

## 2. Methods

### 2.1. Participants

Participants were students recruited through posters and handouts. Potential participants filled out the depression subscale of the Hospital Anxiety and Depression Scale (HADS) [18]. Participants who scored between 4 and 8 and had not previously participated in ABM studies were eligible.

### 2.2. Self-Report Measures

The HADS [18] has a score range of 0–21. A Dutch general population sample had a mean HADS-D score of 3.4, whereas psychiatric outpatients had a mean score of 9.3 [19]. Depressive symptoms were assessed with the Beck Depression Inventory-II (BDI-II). Fourteen is the cutoff score for a mild depression [20]. Anxiety symptoms were measured with the Beck Anxiety Inventory [21].

### 2.3. Dot Probe Task (DPT)

#### 2.3.1. Stimuli

Stimulus pictures were selected from the Karolinska Directed Emotional Faces set [22]. Four separate stimulus sets were created, two containing pictures of happy and neutral expressions and two containing pictures of neutral and sad expressions. Each sets consisted of ten male and ten female actors. The facial expressions were correctly identified at least 60% of the time in ratings obtained from a female student population [23]. The two sets for each emotion (happy, sad) contained different actors and were matched on hit rates, valence, and arousal ratings. Mean hit rates, valence, and arousal ratings for each set are presented in Table 1. A third set of pictures for both emotions was used in the practice trials. All pictures were converted to greyscale and shown against a light grey background. The probe was a small black square (15 × 15 pixels) that was shown either upright (square) or rotated 45 degrees (diamond). Participants were seated circa 60 cm from the display resulting in a horizontal distance of 16.0° of visual angle between the midlines of the two stimulus pictures and the two possible probe locations. The horizontal width of the stimulus pictures and the probe was 8° and 0.4°, respectively.

#### 2.3.2. Single Trial

Throughout the procedure a single trial started with a 750 ms fixation cross-followed by the stimulus images in a horizontal arrangement. Upon offset of stimulus pictures the probe appeared at the location previously taken by either stimulus. The probe remained on the screen until the participant responded by pressing one of two mouse buttons, corresponding to the two possible probe identities (square/diamond). The mouse was fixated to the desk in front of the participant. Upon response a 750 ms intertrial interval preceded the next fixation cross.

#### 2.3.3. Congruency Contingencies

During the pre- and posttraining bias assessment tasks the probe appeared on the location previously occupied by the neutral (incongruent trial) or the emotional stimulus (congruent trial) with equal probability (congruency contingency: 50%). Emotional stimulus location, probe identity, and stimulus identities were randomized and counterbalanced within the congruent and incongruent trials. Within each training session of 200 trials, 170 trials (85%) were either happy congruent (neutral towards happy conditions) or sad incongruent (sad towards neutral conditions). The congruency of 140 of these 170 trials was predetermined. The remaining 30 trials were half of 60 trials with a 50% congruency contingency. These 60 trials were used for the “on-line” assessment of attentional bias within each training session. 

#### 2.3.4. Conditions

Six versions of the dot probe task all differed on two dimensions: direction of training (sad towards neutral or neutral towards happy) and stimulus duration (500 ms, 3000 ms, or variable). Within each block of 40 trials in the variable stimulus duration conditions, the stimulus durations 500, 1125, 1750, 2375, and 3000 ms were randomized and counterbalanced within each combination of trial congruency (for the 50% congruency trials), location of the emotional stimulus, and probe identity. 

### 2.4. Procedure

Participants were scheduled to visit the laboratory on a Monday, Tuesday, Thursday, and Friday within one week. The appointments were scheduled at the same time each day with a maximum deviation of 1.5 hours. Upon completion of the procedure participants were compensated with a small financial reward or course credit. Participants were randomly assigned to one of the six conditions. The Ethics Committee of the Institute of Psychology at Leiden University approved the study, and informed consent was obtained.

The first session started with filling out the questionnaires, followed by 100 trials of a dot probe bias assessment task (congruency contingency of 50%) and 200 trials of the training (congruency contingency of 85%). The second and third sessions consisted of 200 training trials only. The fourth session started with 200 training trials, followed by 100 assessment trials, filling out questionnaires and the debriefing procedure. 

 At the first session participants were required to take as many practice trials as needed for six consecutive correct answers with a minimum of 10 trials. For sessions 2, 3, and 4 only six practice trials with four consecutive correct trials were required. Throughout the experimental sessions short breaks were given every 40 trials; participants could take a few seconds rest and continue the task with a mouse click. After 200 trials in the first and the fourth sessions participants were required to take a 5-minute break. In sessions 1 and 4 the program switched between the pre- and posttraining assessment trials and the training trials without notice.

 Following Wells and Beevers [11], we did not mention the word “training” to the participants, nor any other phrase that implied that their mood might change. Participants were informed that we were interested in the effects of repeated engagement in a task. The debriefing at the end of the last session consisted of a funnelled interview in which participants were first asked to guess the purpose of the study followed by a series of open questions informing whether they noticed anything specific regarding the faces, the location of the faces, the probes, the location of the probes, and whether anything else had changed when the images changed (the switch from the training to the postassessment in the fourth session). Afterwards, the experimenter explained the concept of attentional bias, the dot probe task, and the rationale of the dot probe training. Participants were then told that they could have been in either a training or a control condition (congruency contingency 85% or 50%). All participants had to guess which condition they had been in and rate how certain they were of their answer on a scale ranging from 50 to 100%. This percentage, reversed for those who believed to have been in the control condition, was used as an index of awareness. Finally, participants were fully debriefed and were given the opportunity to ask questions.

## 3. Results

### 3.1. Participants

Two hundred students filled out the HADS-D. Fifty-two of these scored between 4 and 8 points and were subsequently invited. Thirty-three participants were scheduled and engaged in the experimental procedure. Data of three participants had to be discarded due to technical problems. These three participants were replaced to obtain a total of 30 participants. 

### 3.2. Data Preparation and Calculation of Bias Indices

The pre- and posttraining assessment trials as well as the 60 trials with a 50% congruency contingency within each of the four training sessions were included in the analyses. Inspection of the error rates per participant and session showed that none of the participants made more than 10% errors within a single session. Erroneous responses (3% of the dot probe data) were eliminated. Inspection of the remaining data showed that their distribution was skewed, and we therefore used median instead of mean response times to calculate the bias indices [2]. Bias indices were calculated separately for each participant and each of the six phases (pretraining, training sessions 1 to 4, and posttraining) by subtracting the median response time for congruent trials from the median response time for incongruent trials. 

### 3.3. Group Characteristics

Baseline group characteristics are presented in Table 2. Nonparametric tests showed no differences between the groups at baseline (Kruskal-Wallis tests, all *P* > .5).

### 3.4. Change in Bias Index over the Course of Training

**Figure 1:**
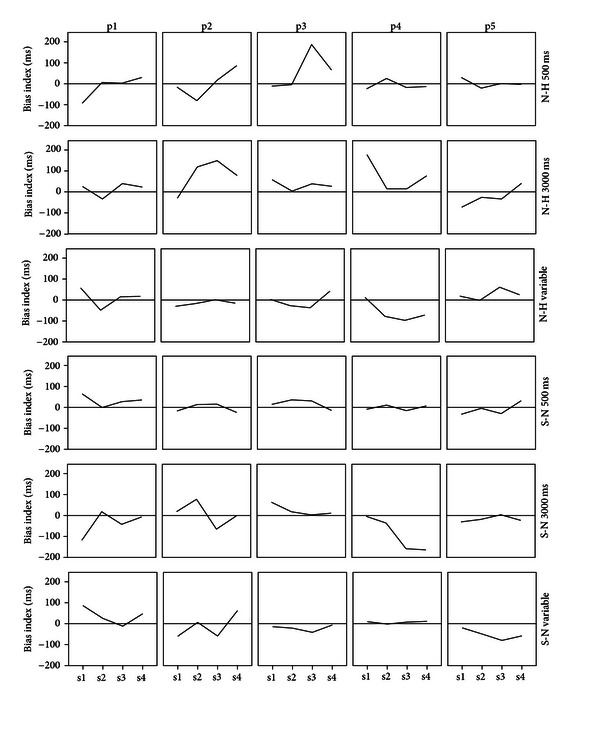
Bias index during the four training sessions for each individual participant. Each row shows the five participants within a condition. Note that for the neutral-towards-happy conditions (top three rows) training should induce an increase in bias index, whereas for the sad-towards-neutral conditions (bottom three rows) training is intended to reduce bias index. N-H = neutral-towards-happy, S-N = sad-towards-neutral, s1 = session 1, and so forth, p1 = participant 1, and so forth.

The bias indices obtained during each of the four sessions for each individual participant are shown in Figure 1. Note that for the upper three rows (neutral-to-happy conditions) an increase in bias index was the intended effect of training, while for the lower three rows (sad-to-neutral conditions) bias index was intended to decrease. 

Visual inspection of each row of panels in Figure 1 learns that none of the training conditions had a consistent effect on the participant's bias indices. The neutral-towards-happy 500 ms condition (top row) had the most consistent effect: relatively large increases in bias indices in three out of five participants, while the other two participant's bias indices remained relatively stable. During the posttraining interview, participant 3 in this condition reported to have noticed at some point that the location of the probe and the happy face may have been associated. The participant then tested and rejected this idea, probably due to the 85% contingency. This may explain this person's peak in bias obtained during the third session. Both 3000 ms conditions (rows 2 and 5) as well as the sad-towards-neutral variable condition (bottom row) showed a change in the intended direction in two participants and a change in the opposing direction in another. The neutral-towards-happy variable condition (row 3) had only one participant who showed the expected change in bias index, while two participants showed a change in the nonintended direction. The neutral-towards-sad 500 ms condition (row 4) had one participant whose bias changed slightly in the nonintended direction, while the other four participant's bias indices remained largely stable. 

### 3.5. Transfer of Training Effects

In order to assess whether a change in bias during the training transferred to a changed bias for the untrained stimulus pictures used in the pre- and posttraining assessment, we calculated two new variables:

(1) SCT(successful  change  trained  stimuli) =bias  index  s4−bias  index  s1,SCU(successful  change  untrained  stimuli) =bias  index  post−bias  index  pre.

SCT and SCU are multiplied by −1 for the sad-towards-neutral conditions so that a positive value of SCT and SCU represents a change in the intended direction, while a negative value represents a change in the nonintended direction. A larger value represents a larger change in bias index at session 4 (SCT) or posttraining (SCU), as compared to session 1 and pre-training, respectively.

**Figure 2:**
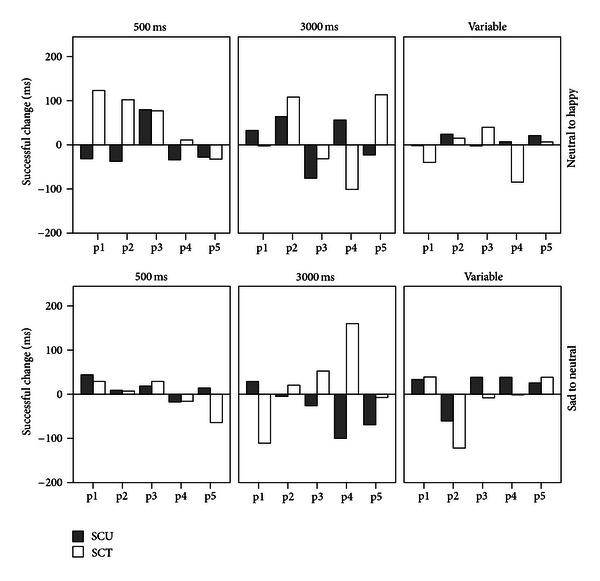
Successful change in bias index for trained (SCT) and untrained (SCU) stimulus pictures per participant ordered by condition. p1 = participant 1, and so forth.

Figure 2 illustrates that changes in bias during the training did not systematically transfer to the untrained stimuli used in the pre- and postassessment. Bias indices of 16 participants changed in opposing directions, while five participants had negative values for both SCT and SCU. Bias index changed in the intended direction for both trained and untrained stimulus pictures in nine participants, as indicated by positive values of SCT and SCU. For one of these participants (p2 in the sad-towards-neutral 500 ms condition) the values of SCT and SCU were 7 and 9 ms, respectively. These values are so small that they likely fall within the range of measurement error. The remaining eight participants whose bias changed in the intended direction for both trained and untrained stimulus pictures were scattered over the six conditions. Three conditions (sad-towards-neutral 500 ms and the two variable ms conditions) each contained two such participants, the neutral-towards-happy 500 and 3000 ms conditions each had one such participant, whereas the sad-towards-neutral 3000 ms condition had no such participants.

### 3.6. Effects on Symptoms

Wilcoxon signed ranks tests comparing the BDI and BAI scores pre- and posttraining for the entire sample indicated no effects of training (*Z* = −1.54, *P* = .12 and *Z* = −1.44, *P* = .15, resp.), as was expected. However, mean BAI scores decreased significantly within the sad-towards-neutral conditions (*Z* = −2.35, *P* = .02) but not within the neutral-towards-happy conditions (*Z* = −.52, *P* = .61). Mean BDI scores did not change significantly within either the sad-towards-neutral or the neutral-towards-happy conditions (*Z* = −1.16, *P* = .24 and *Z* = −1.03, *P* = .30, resp.). 

### 3.7. Awareness of Receiving Training

**Figure 3:**
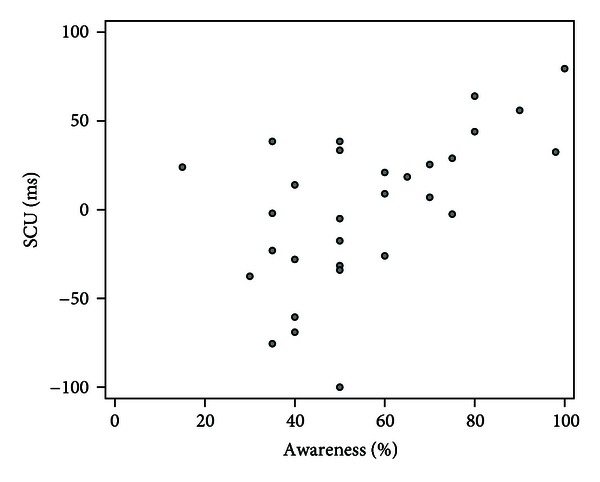
Scatterplot of the correlation between successful change in bias index for untrained stimuli (SCU) and awareness confidence ratings.

At the start of the posttraining funnelled interview, none of the participants accurately guessed the purpose of the experiment. Sixteen participants believed to have been in a (nonexistent) control condition, whereas 14 participants thought to have received training. The confidence with which participants believed to have received training correlated significantly with the change in bias for untrained stimuli (SCU; *r*_*s*_ = .57, *P* = .001) but not with change in bias index for the trained stimuli (SCT; *r*_*s*_ = −.02, *P* = .93) nor with the absolute bias indices obtained during session 4 and the posttraining assessment (*P* = .47 and *P* = .27, resp.). From the scatterplot showing the relation between SCU and the confidence rating (Figure 3) it was observed that this effect was restricted to participants who believed to have received training (confidence ratings > 50%). Indeed, within the group that indicated to have received training, confidence ratings correlated significantly with SCU (*r*_*s*_ = .82, *P* < .001), while the correlation was nonsignificant within the group that believed not to have received training (*r*_*s*_ = −.04, *P* = .89). These correlations differ significantly (test of Fisher z transformed correlation coefficients: *Z* = 2.92, *P* = .004).

## 4. Discussion

We studied six variants of an ABM procedure in a multiple case series design, in order to select the most promising variant for further testing in future controlled clinical trials. No consistent effects on attentional bias were observed for any version of ABM. Changes of attentional bias in individual participants did not generalize to untrained stimuli. This makes it very unlikely that any of these ABM versions will have a specific effect on symptoms in controlled studies. 

During the training, the bias index changed in the intended direction in 17 out of 30 participants. For nine of these 17 participants the bias index for untrained stimulus pictures also changed in the intended direction. This lack of generalization is problematic as generalization of a modified bias is theoretically the key mechanism of ABM. In the study by Wells and Beevers [11] (testing an ABM variant comparable to the current studies' sad-to-neutral 3000 ms condition) for instance, depressive symptoms were unaffected during the training but did improve in the two weeks between training and followup. This is consistent with a model in which cognitive changes only lead to symptomatic changes after interaction with the environment, as has also been proposed for pharmacologically induced cognitive changes [24, 25]. We did not measure longer term effects, but since the effects of training on attentional bias, if any, did not generalize, a delayed effect on symptoms is quite unlikely. Furthermore, 14 of our 30 participants showed a change of bias in the wrong direction.

As expected, depressive symptom levels did not change during the training. Self-reported anxiety symptoms did decrease significantly within the sad-towards-neutral conditions, however. Although the lack of a control treatment makes this finding hard to interpret, we argue that this is a chance finding: an effect on symptoms was not expected, the training was designed to target depression rather than anxiety, and our data do not support the working mechanism that would theoretically mediate such an effect. ABM studies reporting effects on self-reported anxiety measures often do not relate effects to changes in attentional bias, however. 

In an unpublished study comparing implicit and explicit instructions conditions, it was found that explicit instructions increased the effect of ABM on attentional bias but abolished the effect on emotional reactivity [17]. In the current study participants were kept unaware of the study's objectives, but awareness of training contingency was assessed in a funnelled interview following the procedures. One participant reported to have considered the possibility that the probe systematically appeared on the location of one type of stimulus, while 16 out of 30 participants believed to have been in a nonexistent control treatment. The more certain participants were that they had received treatment, the more their bias index for untrained stimuli pictures had changed in the intended direction. No correlation existed between certainty of receiving training and the direction and magnitude of change in bias index during the training. This could be a reflection of demand characteristics in participants who were implicitly aware of the training contingency. It cannot be ruled out that such an effect could also influence self-reported measures of symptoms. Further research into the role of awareness in ABM seems warranted.

In summary, this single case series approach enabled us to scrutinize the effects of six variants of ABM for depression at the individual's and at training session levels. Since we did not observe any consistent effects on attentional bias, we advice not to proceed yet to RCTs to test DPT-based ABM for depression. Given the large placebo effect in clinical trials of affective disorders, the risk of nonreplicable findings would be large. 

## Figures and Tables

**Table 1:** Hit rates, intensity, and arousal ratings for each of the four stimulus sets.

Set	Emotional pictures			Neutral pictures		
	Hit rate	Intensity	Arousal	Hit rate	Intensity	Arousal
Happy A	78.8 (10.8)	6.1 (.9)	3.8 (.4)	78.8 (10.8)	5.0 (.5)	2.5 (.2)
Happy B	78.6 (10.9)	6.2 (.6)	3.7 (.4)	78.6 (10.9)	4.9 (.5)	2.6 (.2)
Sad A	84.6 (10.1)	5.5 (.7)	3.5 (.4)	72.5 (12.8)	4.9 (.5)	2.6 (.2)
Sad B	84.8 (10.3)	5.2 (.9)	3.3 (.5)	80.9 (11.3)	5.0 (.5)	2.5 (.2)

^a^All measures are reported as *M* (sd).

^b^Based on ratings provided by Goeleven et al. [23].

**Table 2:** Characteristics at baseline, per condition.

	Sad towards neutral			Neutral towards happy		
	500 ms	3000 ms	Variable	500 ms	3000 ms	Variable
Age	24.0 (7.9)	19.4 (1.5)	20.2 (1.5)	20.0 (1.4)	20.6 (5.3)	21.0 (3.3)
BDI-II	11.4 (4.7)	13.8 (7.7)	12.6 (6.4)	14.8 (4.7)	11.8 (6.3)	15.2 (5.5)
BAI	13.4 (5.9)	11.0 (5.6)	9.2 (5.8)	11.4 (5.5)	10.0 (4.5)	13.2 (4.8)

^a^All measures are reported as *M* (sd).

^b^BDI-II: Beck Depression Inventory II; BAI: Beck Anxiety Inventory.
